# Digital Training for Non-Specialist Health Workers to Deliver a Brief Psychological Treatment for Depression in Primary Care in India: Findings from a Randomized Pilot Study

**DOI:** 10.3390/ijerph17176368

**Published:** 2020-09-01

**Authors:** Shital S. Muke, Deepak Tugnawat, Udita Joshi, Aditya Anand, Azaz Khan, Ritu Shrivastava, Abhishek Singh, Juliana L. Restivo, Anant Bhan, Vikram Patel, John A. Naslund

**Affiliations:** 1 Sangath, 120 Deepak Society, Chuna Bhatti, Kolar Road, Bhopal 462016, India; shital.muke@sangath.in (S.S.M.); deepak.tugnawat@sangath.in (D.T.); udita.joshi@sangath.in (U.J.); aditya.a@sangath.in (A.A.); azaz.khan@sangath.in (A.K.); ritu.shrivastava@sangath.in (R.S.); abhishek.singh@sangath.in (A.S.); anant.bhan@sangath.in (A.B.); 2 Department of Global Health and Social Medicine, Harvard Medical School, Boston, MA 02115, USA; Juliana_Restivo@hms.harvard.edu (J.L.R.); vikram_patel@hms.harvard.edu (V.P.); 3 Department of Global Health and Population, Harvard TH Chan School of Public Health, Boston, MA 02115, USA

**Keywords:** depression, psychological treatment, task sharing, primary care, pilot study, non-specialist health worker, training, digital technology, mental health

## Abstract

*Introduction*: Task sharing holds promise for scaling up depression care in countries such as India, yet requires training large numbers of non-specialist health workers. This pilot trial evaluated the feasibility and acceptability of a digital program for training non-specialist health workers to deliver a brief psychological treatment for depression. *Methods*: Participants were non-specialist health workers recruited from primary care facilities in Sehore, a rural district in Madhya Pradesh, India. A three-arm randomized controlled trial design was used, comparing digital training alone (DGT) to digital training with remote support (DGT+), and conventional face-to-face training. The primary outcome was the feasibility and acceptability of digital training programs. Preliminary effectiveness was explored as changes in competency outcomes, assessed using a self-reported measure covering the specific knowledge and skills required to deliver the brief psychological treatment for depression. Outcomes were collected at pre-training and post-training. *Results*: Of 42 non-specialist health workers randomized to the training programs, 36 including 10 (72%) in face-to-face, 12 (86%) in DGT, and 14 (100%) in DGT+ arms started the training. Among these participants, 27 (64%) completed the training, with 8 (57%) in face-to-face, 8 (57%) in DGT, and 11 (79%) in DGT+. The addition of remote telephone support appeared to improve completion rates for DGT+ participants. The competency outcome improved across all groups, with no significant between-group differences. However, face-to-face and DGT+ participants showed greater improvement compared to DGT alone. There were numerous technical challenges with the digital training program such as poor connectivity, smartphone app not loading, and difficulty navigating the course content—issues that were further emphasized in follow-up focus group discussions with participants. Feedback and recommendations collected from participants informed further modifications and refinements to the training programs in preparation for a forthcoming large-scale effectiveness trial. *Conclusions*: This study adds to mounting efforts aimed at leveraging digital technology to increase the availability of evidence-based mental health services in primary care settings in low-resource settings.

## 1. Introduction

According to the global burden of disease study, nearly 200 million people were living with mental disorders in India by 2017, which represents 14.3% of the total population of the country [1]. This includes over 45 million people living with depressive disorder, the leading mental health contributor to the global disease burden, comprising approximately 3.3% of the total population of the country [1]. The National Mental Health Survey of India 2015–16 found that the prevalence of depression was about 2.7% and the lifetime prevalence was 5.3% in the study population [2]. Several studies have reported a significant gap between those living with depression and those who have access to adequate care [3,4]. The National Mental Health Survey estimated that the care gap for current depression was 79.1% [2], while in some regions of the country this gap exceeds 90% [5].

The World Health Organization’s (WHO) Mental Health Gap Action Programme (mhGAP) recommends brief psychological interventions as first-line treatments for depression [6]. However, access to brief psychological treatments remains a significant challenge, particularly in lower-income countries like India. This is partly due to the limited number of available specialist providers to deliver these treatments or supervise care, as well as to train other therapists [7,8]. Task sharing involves building the capacity of non-specialist health workers, which include a broad range of frontline health workers who do not have specialized training in mental health care, to deliver brief evidence-based psychological treatments for common mental disorders [9]. This approach appears to be a key strategy to address the care gap for depression, as reflected by mounting evidence that non-specialist health workers can effectively deliver brief psychological treatments for depression across a range of lower resource settings [10,11,12].

In India, the formation of the National Mental Health Policy of India in 2014 [13] and enforcement of the Mental Health Care Act 2017 [14], as well as revised guidelines of the National Mental Health Program (NMHP) [15], are major drivers at the policy and health system level for expanding and integrating mental health services in primary health care [16]. These recent legislative developments have highlighted the importance of task sharing as being critical to achieving universal coverage of basic mental health services. A major barrier to the successful implementation and scale up of task sharing is the need to adequately train sufficient non-specialist health workers to deliver brief psychological treatments and to ensure that this workforce achieves the necessary clinical competencies to sustain delivery of high-quality care [17,18,19].

In India, conventional face-to-face residential training requiring extended stays at government training facilities is the typical approach for training non-specialist health workers, such as ASHAs (Accredited Social Health Activists) through the National Health Mission [20,21,22,23]. However, there are financial and logistical challenges, such as the need for expert trainers to lead the training, as well as the requirement of significant travel across long distances for participants to attend the training [7,24]. Therefore, this method of training health workers is limited by poor scalability. The increasing availability and use of digital technologies, such as smartphones, among non-specialist health workers offer new opportunities to support training and skill-building without requiring in-person instruction [25,26]. For instance, mobile internet penetration continues to increase rapidly in many low-resource countries, with close to 450 million people in 2020 in India having internet access from their phones [27]. While many frontline health workers do not have access to or own smartphones, this is changing in several parts of India as government health systems are now providing smartphones to health workers to support them in their work [28,29].

To date, there have been promising initial efforts demonstrating the feasibility of using digital technology as a tool for enhancing in-person training programs for non-specialist health workers in a low-resource setting in rural Pakistan [30]. Additionally, prior studies have demonstrated promising findings using digital technology to support task-sharing mental health services in low-resource settings through the use of digital tools for diagnosis, guiding clinical decision making, and facilitating supervision [25]. Specifically in India, recent studies have reported on a successful digital decision-support platform for supporting community health workers and primary care providers in the screening, diagnosis, and management of common mental disorders [31]; the use of an Android app with tailored video content for training community volunteers about mental health, connecting individuals with available services, and raising awareness [32]; and the initial feasibility and acceptability of a digital game accessible from a smartphone app involving a problem-solving intervention for adolescent mental health [33]. These studies highlight the viability and promise of digital interventions for mental health in low-resource settings such as India; however, there remains an immediate need to generate evidence on the feasibility, acceptability, and potential effectiveness of using a fully remote digital training program delivered on a smartphone application to non-specialist health workers in a rural area of a low-resource setting.

In earlier formative research, we demonstrated the interest in using digital technology for accessing a training program to deliver a brief psychological treatment for depression among non-specialist health workers in Madhya Pradesh, India [34]. We found that a digital platform was feasible for use among non-specialist health workers, and through a series of focus group discussions, we gained valuable stakeholder insights about what features could make a digital training program interesting and appealing for this target group. Specifically, participants provided suggestions for simplifying the language in the program contents and materials, and using familiar terms tailored to the local context; they also recommended adding more images or graphics and interactive features to create a more engaging training program [34]. Drawing from these findings, our team developed a digital program for training non-specialist health workers to deliver the Healthy Activity Program (HAP), a brief evidence-based psychological treatment for depression in primary care [35].

Our next step, and primary objective of this pilot study, was to determine the feasibility and acceptability of this digital training program compared to conventional face-to-face training. In this pilot study, our goal was to collect data on the use of the digital training, such as navigating the smartphone app and accessing the training content, as well as participant feedback to inform refinements to the digital training as well as our study procedures in preparation for a larger fully powered effectiveness trial. Specifically, we conducted this three-arm randomized pilot trial to explore the acceptability and feasibility of two digital training programs (digital training alone and digital training with remote support), and to explore changes in competency outcomes compared to conventional face-to-face training for non-specialist health workers, to deliver the evidence-based HAP treatment for depression in Sehore, a rural district in Madhya Pradesh, India.

We included a third arm in this pilot study to test the use of remote support for enhancing engagement and completion of the digital training program. Our rationale for using remote support stems from the existing online education literature highlighting that additional support can promote participant engagement and completion in online learning programs [36,37]. While this study was primarily focused on determining the feasibility and acceptability of the digital training program, we also collected a measure of competency to assess preliminary effectiveness, which was defined by the acquisition of the knowledge and skills required to deliver HAP.

## 2. Material and Methods

This exploratory three-arm randomized pilot study followed the extension of CONSORT guidelines to pilot studies [38]. In this study, non-specialist health workers were recruited from three community health centers (i.e., Doraha, Bilkishganj, and Shyampur) in the Sehore district of Madhya Pradesh, India. This study site was selected because Sangath, the research organization leading this project, has a close partnership and an established Memorandum of Understanding with the state government. Additionally, the goal was to create a model of depression care that could be successfully delivered by non-specialist health workers in Sehore district and then scaled up to other districts in the state, and also to other regions of India. Madhya Pradesh is a large, centrally located state with over 72 million people, of which nearly 73% reside in rural areas [39]. Relative to many other Indian states, Madhya Pradesh ranks lower with respect to human development and availability of resources [40,41]. According to the 2016 National Mental Health Survey of India, the care gap for mental disorders in Madhya Pradesh exceeds 90% [5]. Ethics review boards at Sangath, India (VP_2017_028), and Harvard Medical School, USA (IRB17-0092), approved all study procedures.

### 2.1. Sample

The target sample for this pilot trial was 45 non-specialist health workers. This sample size was considered sufficient for achieving our primary goal of assessing acceptability and feasibility of the training programs [42] and was also selected to ensure we had the minimum number of participants for in-person instruction in the face-to-face training (*n* = 15). The sample included Accredited Social Health Activists (ASHAs), ASHA Facilitators and Multi-Purpose Health Workers (MPWs) employed in the National Health Mission (Madhya Pradesh state) in India. ASHAs are all women, and a cadre of community health workers in India, introduced by the National Health Mission (NHM) in 2005 with the goal to serve as health activists in the community, create awareness on health and its social determinants, as well as to mobilize the community especially marginalized populations to increase utilization and accountability of the existing health services [43]. Each ASHA covers a population of 1000 and receives performance-based and service-based incentives as compensation for facilitating immunization, referral, and escort services for institutional deliveries [44]. ASHA Facilitators work as a support mechanism to ASHAs to provide mentoring and support and to monitor performance. One ASHA Facilitator typically works with 10 to 20 ASHAs [43]. MPWs are male health workers who are appointed primarily for the control of communicable diseases and are a key functionary at Sub-Health Centers, which are the most peripheral health facilities covering a population of 5000 to deliver preventative health services to the community [43].

Eligible non-specialist health workers who met the inclusion criteria of having age ≥18 years (the minimum age required for employment by the National Health Mission [22,45]); being a certified non-specialist health worker (i.e., ASHA, ASHA Facilitator, or MPW); having a minimum education level of 8th standard (i.e., to ensure they can read and write to access the digital program, written training materials, and complete study assessments); willing to provide written informed consent; and, willing to complete the full training program and stay in the study area during the pilot trial period. Non-specialist health workers were excluded if they had participated in prior formative research activities conducted by our research team (due to prior exposure to the training content), which we confirmed by referring to the list of non-specialist health workers provided by a National Health Mission official in the district (see below), or if they had significant speech, sight, or hearing impairment, or were unable to read or write. Smartphone ownership was not required to participate, as participants were provided with a smartphone to access the digital training programs.

### 2.2. Recruitment Procedure

A district National Health Mission official provided the list of non-specialist health workers from the three community health centers. Community health centers represent secondary level health services facilities designed to provide referrals as well as specialist care to rural populations [46,47]. We screened the list containing 377 health workers based on the criteria of age (18 years and above), education (8th standard and above), and non-participation in earlier formative research activities. We found a total of 302 potentially eligible health workers. From this list, our data manager randomly selected 92 potentially eligible health workers using the Research Electronic Data Capture (REDCap) software [48]. Research assistants then contacted these potentially eligible health workers by phone to confirm their interest and availability to participate in this pilot study.

Potentially eligible non-specialist health workers were invited to attend a group information session at a nearby community health center to learn more about the study. The research team organized the group information session to describe the purpose of the study and to inform participants that this study involves collaboration with the National Health Mission. The information session also served as a way to explain what the training involves, the study procedures, and the pre- and post-training assessments. This was also an opportunity for participants to ask questions about the study, and to emphasize that their decision to participate was completely voluntary. After the group information session, non-specialist health workers who expressed interest in participating confirmed their eligibility criteria (age and education), and were provided with an information sheet and completed individual written-informed consent. During the individual consent process, health workers were informed that their decision to participate or withdraw from the study at any time would not have any adverse consequences on their current standing as a health worker or their employment status, and that any data collected during the study would be kept confidential and that no identifiable results would be shared with the health system or others outside the research team.

### 2.3. Randomization

Participants were randomly allocated to one of three training programs based on stratification variables of age, education, and type of non-specialist health worker (i.e., ASHA, ASHA Facilitator, MPW). The study data manager conducted the randomization using the randomizer package available in R software. The age range of recruited ASHAs (24–42 years), ASHA Facilitators (31–46 years), and MPWs (39–52 years) varied widely, hence it was decided to keep different cut-off points for stratification based on the average age for each category of health worker. Two strata for age variables for ASHAs were age ≤35 and age >35; for ASHA Facilitators were age ≤37 and age >37; and for MPWs were age ≤47 and age >47. Similarly, stratification for education for ASHAs was 8th standard and >8th standard; for ASHA Facilitators was 8–12th standard and >12th standard; and for MPWs was 8–12th standard and >12th standard. In total, there were 12 strata to maintain a balance of participant characteristics across study arms. This also served as an opportunity to pilot test our randomization procedures in preparation for the forthcoming larger trial.

### 2.4. Training Programs

The training programs in this study were designed to provide instruction to non-specialist health workers to gain the clinical skills and competencies necessary to deliver the Healthy Activity Program (HAP) for treatment of depression [49]. The HAP is an evidence-based brief psychological treatment for depression designed and tested in Goa, India, that has demonstrated effectiveness and cost-effectiveness in primary care settings in India [35], as well as sustained clinical benefits [50]. The success of the HAP for treating depression has also been demonstrated in other lower-resource contexts, including among people receiving treatment for multidrug-resistant tuberculosis [51] and people with severe depression in primary care settings in Nepal [52], and as part of recent efforts to scale up mental health services in Madhya Pradesh [53]. The HAP consists of two manuals covering general counseling skills and treatment specific skills. These manuals are open source and available online (http://www.sangath.in/). These manuals were adapted to the local context of Madhya Pradesh and converted into digital and F2F training programs (i.e., covering the same content using different teaching strategies). In this pilot study, we compared three different training programs: conventional face-to-face (F2F) training; digital training (DGT); and digital training with remote support (DGT+).

#### 2.4.1. Conventional Face-to-Face Training (F2F)

The conventional F2F training consists of a six-day classroom training facilitated by two experienced counselors with certification as Master Trainers, meaning that they have significant experience delivering the HAP to patients with depression in clinical settings, have completed instruction in being an effective trainer, and have provided training to other health workers in the delivery of the HAP. This conventional in-person training is considered the ‘gold-standard’ in training non-specialist health workers based on the prior methods employed in the evaluation and delivery of the HAP treatment [35]. The six-day training is hosted in a community setting and follows the content in the HAP manuals. This form of in-person training is consistent with the type of training currently available to non-specialist health workers in the district.

#### 2.4.2. Digital Training (DGT)

DGT consists of a digitized version of the HAP manuals hosted on the Moodle Learning Management System and accessible through a smartphone app [54]. The training program content was divided across 16 modules following the same structure as the F2F instruction. The modules consisted of expert lecture videos, role-play videos showing clinical scenarios, PowerPoint presentations, reading materials, interactive quizzes embedded within the modules, and assessment questions at the end of each module. Duration of digital training content was matched to the duration of the F2F training, and consisted of approximately 48 h, covering the time required to view the content, read the accompanying materials, and complete the interactive quizzes and assessment questions. Participants were provided with a smartphone to access the training program and were invited to attend a short orientation session to learn how to use the phone, access the instructional content through the smartphone app, and navigate the Learning Management System interface. Participants were provided with a 30-day window to complete the digital training. Throughout the training, participants could contact our research team for technical assistance regarding any concerns or challenges with using the smartphone or accessing the training program content.

#### 2.4.3. Digital Training with Remote Support (DGT+)

The DGT+ training program includes access to the same smartphone app, digital training content, and technical support described above for the DGT program. DGT+ included the addition of remote support from the research team. This involved weekly phone calls from a research assistant to participants. The purpose of the support phone calls was to check in with participants about their progress with the training, and whether they had experienced any challenges or had questions about the digital platform or program content. The research assistant also provided participants with encouragement and praise during the calls as a way to motivate participants and support engagement in the training.

### 2.5. Outcome Assessment

We collected outcomes on acceptability and feasibility of the training programs and preliminary effectiveness of the training on competency outcomes. After informed consent, a unique participant ID number was assigned to each participant. This number was used on all subsequent data collection forms, with no participant name or identifiable information used on any of the data collection forms. Prior to the outcome assessment, participants were informed of the purpose of using participant ID numbers for their identification throughout the study duration, rather than using their names. Study outcomes were collected before and after the training using paper-based forms distributed in-person at the community health center. The average duration of completing the outcome assessment was approximately 2 h. We used paper-based forms instead of digital data collection to avoid giving an unfair advantage to participants in the digital training programs, as the F2F training participants may not have had equivalent exposure to use of digital technology. Members of our research team who were blind to arm allocation, and who were not involved in the development of the training programs, collected the outcome assessments.

#### 2.5.1. Acceptability and Feasibility Indicators

We collected process indicators to determine participant engagement and use of each of the training programs. This included: daily attendance at the F2F training; metrics collected from the Learning Management System for the digital training programs (for both the DGT and DGT+ programs), including the number of days to complete the training program and the number of modules completed; the number of phone calls made by the participants for seeking technical assistance (for both the DGT and DGT+ programs) from the research team; and the number of phone calls initiated by the research team to participants for follow up with their queries and types of challenges or questions that commonly were mentioned (for the DGT+ only).

After completing the training (end line), we also collected a satisfaction and acceptability questionnaire adapted from an existing measure of motivation and engagement in face-to-face and online education programs called the MUSIC^®^ model of motivation inventory [55,56,57,58]. The 26-item questionnaire asks about the level of satisfaction with the training, acceptability of the content and method of instruction, and feasibility of completing the training. The items are rated on a six-point Likert scale, and relate to feasibility, acceptability, adoption, and appropriateness of the training programs. The questionnaire was translated into Hindi and modified for the F2F and digital (DGT and DGT+) training programs. The items are rated on a six-point Likert scale, with 1 being the lowest and 6 the highest score. The questionnaire covers the domains of acceptability, appropriateness, adoption, and feasibility. The average score of each domain was calculated by adding the score of all the items in the domain divided by the number of questions in the domain.

We also conducted one focus group discussion for all the participants in each arm to obtain feedback about the training and to ask questions pertaining to acceptability and appropriateness of the content, methods of instruction, and engagement in the training, as well as the feasibility of accessing and navigating the digital training platform on the smartphone app, and providing recommendations for what could be improved in the training program. The focus group discussions lasted about 45–60 min, were facilitated by a qualitative researcher and were audio-recorded for analysis. Another researcher from our team observed the focus groups to collect field notes to identify key recommendations from participants for modifying the training programs.

#### 2.5.2. Preliminary Effectiveness Outcome

Competency outcomes were collected before (baseline) and after (end line) the training to determine the preliminary effectiveness of the different training programs. Competency was assessed using a questionnaire consisting of short clinical vignettes followed by multiple-choice questions covering the core skills and competencies needed to deliver the HAP. The measure was based on prior research showing that self-assessment can reliably assess therapist competency following training [24,59,60]. Three equivalent versions of the questionnaire were used to allow repeat testing. The measure focuses on testing knowledge of the HAP treatment as well as applied knowledge of how to deliver the treatment, an essential aspect of provider competence. This questionnaire is scored from 0 to 100, with higher scores reflecting higher levels of knowledge and competency. The questionnaire was translated into Hindi for this study, and modifications were made to fit the local context, such as simplifying complex or technical language and using local terms. Experienced counselors reviewed the Hindi translation to ensure that it was appropriate for administering to non-specialist health workers in the local context in Madhya Pradesh. To avoid “teaching to the test” [61], none of the individuals involved in the development of the HAP training materials had access to the competency assessment questionnaire.

### 2.6. Data Analysis

Descriptive statistics were computed for socio-demographic characteristics between the three training programs. Process indicators and the satisfaction questionnaires were summarized in tables. Field notes were collected during the focus group discussions to capture key feedback for supporting refinements to the programs. As this was a pilot study with the primary goal of determining feasibility and acceptability of the training programs and to inform improvements to the instructional content and delivery of the training programs, we did not conduct an in-depth thematic analysis of the qualitative data. Rather, we followed guidance from the person-based approach to intervention development [33,62], which enabled the combination of quantitative and qualitative data to inform modifications to the training programs. Specifically, we used a framework analysis approach [63] to guide our identification of common topics within the qualitative data, following a coding framework with the four core domains outlined from the satisfaction and acceptability questionnaire (i.e., appropriateness, acceptability, adoption, and feasibility) [55]. One researcher from our team who was not involved in the development of the training programs coded the transcripts following this *a priori* framework and categorized key observations from participants according to each of the broad domains. Two additional researchers from our team who supported the development of the training programs reviewed the classification of participants’ observations and the key recommendations for improving the program. This second round of review provided an opportunity to expand on any observations that were not clear, and to draw from the field notes to supplement the description of the recommendations. A fourth researcher who was external to this process then reviewed the tables summarizing the qualitative feedback to ensure that actionable steps could be identified for improving the usability and acceptability of the training programs in preparation for a subsequent large scale randomized controlled effectiveness study.

As part of an exploratory analysis of change in the competency assessment outcome, we used a paired *t*-test to determine if there was a statistically significant mean difference between the competency scores obtained before and after the training. We also explored pre- and post-training differences in the competency assessment scores within the three training programs using a non-parametric Wilcoxon signed-rank test [64]. This method was selected to account for the small sample size. Due to the heteroscedasticity, since the *p*-value for the Bartletts’s test for homogeneity of variance was less than 0.05, we used Welch’s one-way ANOVA test to determine if the change in competency assessment scores obtained before and after the training program was different for the three arms, followed with a Games–Howell post-hoc test. All analyses were completed using STATA (StataCorp LLC, College Station, TX, USA), and *p* < 0.05 was considered statistically significant.

## 3. Results

**Figure 1:**
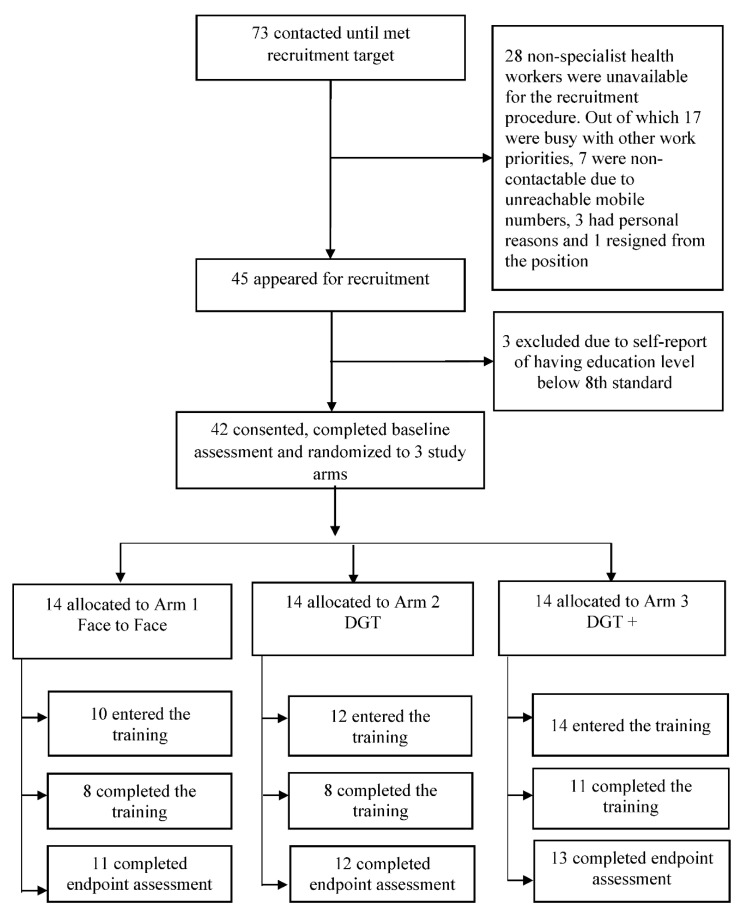
Participant flow diagram. DGT: Digital Training.

Out of 92 potentially eligible non-specialist health workers, we contacted a total of 73 until reaching our recruitment target of 45. These 45 non-specialist health workers were invited to attend the group information session to learn more about the study. As outlined in Figure 1, 42 consented and enrolled in the study and were randomly allocated to the three study arms. This included 23 ASHAs, 10 ASHA Facilitators, and 9 MPWs. Participant characteristics are summarized in Table 1. Of the 42 enrolled participants, 36 started the training programs to which they were randomized (*n* = 10 in F2F; *n* = 12 in DGT; *n* = 14 in DGT+) and 36 (86%) participants completed post-training assessments (*n* = 11 in F2F; *n* = 12 in DGT; *n* = 13 in DGT+). We found that there were no differences in participant baseline characteristics (such as type of health worker, mean age, education, and gender) between those who completed the training compared with those who did not complete the training. No harms were recorded for any participants throughout the duration of this pilot study.

### 3.1. Acceptability and Feasibility Indicators

Process indicators are listed in Table 2. Six participants never started the training program, out of which 5 were the MPWs and 1 was an ASHA. The reasons for not starting the training were largely due to other work commitments, and other personal or family commitments. Further, several participants (*n* = 9) started the training but could not complete it. This was similarly due to other family or work commitments, and inclement weather as the training happened during the monsoon season (making it difficult to travel to the training facility for F2F participants). Thus, 27 (64%) participants completed the full training program, with 8 (57%) in F2F, 8 (57%) in DGT, and 11 (79%) in DGT+. We observed differences in program completion between the different types of non-specialist health workers, where 16 (70%) ASHAs, 8 (80%) ASHA Facilitators, and 3 (33%) MPWs completed the training.

There were a total of 399 support calls related to technical assistance for the digital training programs. Among the DGT participants, there were 255 calls. This involved calls made by the participants and calls made by the research team to respond to the participants. In total, 58% of the calls (149 out of 255) were from participants to the research team. While 42% of the calls (106 out of 255) were from the research team in response to participants’ queries. For DGT+ participants, the major difference was that our research team initiated the calls (as opposed to participants initiating calls). Among DGT+ participants, there were 144 calls. In total, our research team initiated 60% of the calls (87 out of 144) to participants, while 40% of the calls (57 out of 144) were from participants to our research team. The number of calls per participant ranged from 4 to 37. The calls primarily related to technical challenges, as summarized in Table 3, such as poor connectivity, the mobile app not loading or being deleted from the phone, and challenges with navigating the course content.

Table 4 summarizes participants’ responses to the satisfaction and acceptability questionnaire for each training program. Mean score across the domains was generally 5 or greater (out of a possible score of 6), indicating that participants rated the training programs favorably for feasibility, acceptability, and adoption. Across study arms, appropriateness was ranked lowest, suggesting that additional efforts are necessary to promote engagement with the program content. Findings from the focus group discussions (*n* = 28 participants) were grouped within the same four domains from the satisfaction and acceptability questionnaire, as highlighted in Table 5. Recommendations for improving the F2F training including increasing the duration of the training and clarifying some of the training manual content. For the digital training programs, the main recommendations were related to ensuring that the entire course could be accessed offline due to poor internet connectivity in the region, as well as providing a more comprehensive orientation session at the beginning of the program to provide an overview of the smartphone app and navigating the digital program interface, as well as extending the availability of telephone support from the research team.

### 3.2. Preliminary Effectiveness Outcome

**Figure 2:**
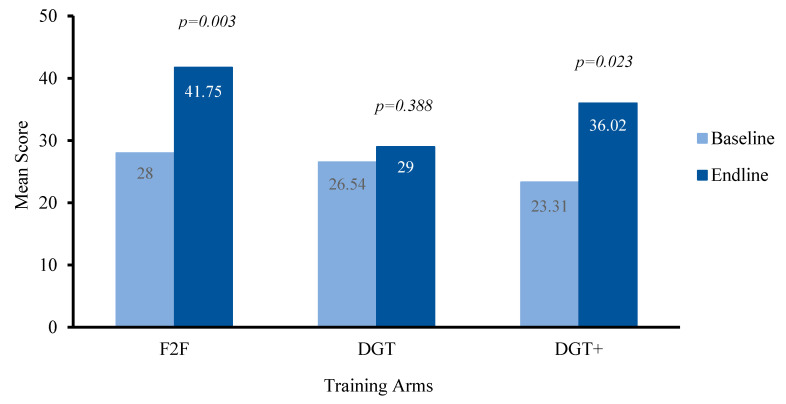
Change in competency assessment scores within each training program. Note: this Figure includes scores from the *n* = 11 in F2F, *n* = 12 in DGT, and *n* = 13 DGT+ participants who completed the post-training (endline) competency measure; though, some of these participants did not complete the training programs. F2F: Face-to-Face; DGT: Digital Training.

Using a paired *t*-test to explore whether there was a statistically significant mean difference between the competency scores obtained pre- and post-training for all participants (all three training programs combined), we found that participants (*N* = 36) overall scored better on the post-training assessment (Mean = 35.43; SD = 11.39) compared to the pre-training (baseline) assessment (Mean = 25.82; SD = 7.42), with a maximum attainable score of 100. This represents a significant increase of 9.61 points (95% CI: 5.17 to 14.04), *t* (35) = 4.401, *p* < 0.0005, suggesting that competency scores increased after completing the training program regardless of training format (F2F or digital). For the F2F training, the Wilcoxon signed-rank test showed a significant change in participants’ competency scores (*Z* = 2.934, *p* = 0.0033). For the DGT training participants, the change was not significant (*Z* = 0.863, *p* = 0.3882), whereas, for the DGT+ training participants, the change was statistically significant (*Z* = 2.271, *p* = 0.0231), as illustrated in Figure 2. For F2F, the mean competency score improved by 13.8 (SD = 6.6) points, while for the DGT and DGT+ arms it was 2.5 (SD = 7.8) points and 12.7 (SD = 18.2) points, respectively.

Next, we explored changes in scores on the competency measure between training groups. We conducted a Welch’s ANOVA test, which showed that there was a statistically significant difference in change in the competency score obtained before and after the training between the three groups, *F* (2,21) = 7.0358, *p* = 0.00455. Following up with a Games–Howell post-hoc test, we found that there was a statistically significant difference in the scores on the competency assessment obtained pre- and post-training between the F2F and DGT arm with *p* < 0.01, but not between the F2F and DGT+ arms.

## 4. Discussion

This pilot study evaluated the feasibility and acceptability of conventional F2F training compared with digital training programs to build the capacity of non-specialist health workers for delivering HAP, a brief evidence-based psychological treatment for depression. The primary goals of this study were to test the study procedures and, importantly, to inform modifications and refinements to the training programs in preparation for a large-scale fully powered effectiveness trial. While we previously demonstrated the interest in using digital technology for accessing training programs among non-specialist health workers, the current study substantially expanded on our prior work by testing these programs in the field, allowing the opportunity to gain insights about the use of the training programs in real world settings.

This study highlighted the need for several significant modifications to the digital training program. These included: the need to modify the timing and structure of the F2F training to accommodate participants’ long commutes from distant villages, as well as to account for their family responsibilities such as childcare; the need to ensure that the digital program content could be accessed entirely offline given the low internet connectivity in rural areas in the Sehore district; the need for a more comprehensive orientation session for using the smartphone app to access the training program and navigating the Learning Management System, including use of more pictures and screenshots of ‘how to’ examples to account for low digital literacy among participants (this was also reflected by the large number of technical assistance calls received during this pilot study); and modifications to the provision of technical support, to allow early identification of participants who may be struggling to complete the digital training and enable a more timely response to technical challenges that could arise. The challenge of poor internet connectivity was similarly reported in a prior trial from Pakistan, where efforts to address this concern also involved ensuring access to the training content in an offline format [30]. This prior study reported that the online training approach required a stable internet connection that may not be available in many remote, rural, resource-poor settings; hence, to increase the feasibility of the online training program, the researchers used an offline tablet-based application to deliver the training to frontline health workers [30]. Following the focus group discussions, we made substantial modifications to the remote support component of the DGT+ arm, given that participants expressed high interest in having a member of our research team contact them to provide encouragement and motivation on a regular basis.

Our study aligns with an emphasis in the digital mental health research literature that it is necessary to consider the perspectives of users in order to support adoption, engagement, and sustained use of digital interventions [65,66,67]. Despite the large number of technical challenges that participants mentioned throughout this pilot study, we were reassured by participants’ continued interest in learning about the mental health treatment related content. This was consistent between participants in the digital training programs and F2F training, suggesting recognition among non-specialist health workers of the importance of depression care in their communities. This is an essential first step towards successfully scaling up mental health services in primary care settings in the Sehore district, as well as across the state and nation.

Another important finding in this study was about which cadre of non-specialist health workers would be most suitable for completing the training program based on their availability. We learned that the MPWs had too many other competing demands, and were frequently called away by their superiors for attending to urgent duties, which is reflected in their low completion rates across the three training programs. However, for non-specialist health workers who are frequently required to travel for other work related activities, use of a digital training program may offer the opportunity for these individuals to gain the necessary skills to deliver mental health services while accommodating their already busy workload. In addition, digital training holds the potential to train all types of health workers on depression care, as the program can be accessed on a smartphone, which could potentially expand access to mental health services at the community level, thereby advancing efforts to achieve the Mental Health Care Policy goal of providing universal mental health care services for all [13].

While this was a pilot study primarily focused on assessing the feasibility and acceptability of the different training programs, we found that scores on the competency assessment improved for the F2F and DGT+ participants. This is a promising finding, suggesting that digital training with added support may be equally effective compared to the regular classroom or in-person training in terms of gaining knowledge and skills. Also, digital training is potentially more convenient, feasible, and scalable for building the capacity of non-specialist health workers when compared to conventional in-person training, which is supported by similar studies and recent reviews from other low-income and middle-income countries [30,68,69]. Additionally, the findings also indicate that the training content is appropriate for gaining the knowledge and skills related to HAP delivery, as reflected by improved scores on the competency measure. However, the DGT participants did not show significant improvements, suggesting that the use of digital technology alone may not be sufficient for contributing to knowledge acquisition. Importantly, the addition of support initiated by our research team appeared to greatly improve program completion for DGT+ participants (79%) compared to DGT participants (57%). This is consistent with prior studies of online education programs that have demonstrated that the use of digital training is most effective when supplemented with access to remote or in-person support [30,36]. For example, the recent study of the Technology-Assisted Cascaded Training and Supervision system for Lady Health Workers, conducted in rural areas in Pakistan, found that use of digital technology in combination with in-person support and training contributed to comparable improvements in competence as conventional face-to-face training [30]. Additionally, a study in Zambia using technology to train community health workers highlighted a similar finding that in-person support is required to address technical challenges related to poor network coverage, mobile hardware, and software [70]. If these challenges are not addressed, it can negatively impact the delivery process and training outcomes [70]. Further, a recent review of mobile technologies for education and the training of community health workers in low-income and middle-income countries indicated the value of digital training methods for augmenting periodic in-person training activities, while highlighting that digital training programs could be embedded within existing health care services to allow opportunities for continuing education among community health workers [69].

Several limitations with this study warrant consideration. Firstly, this was a pilot study looking at acceptability and feasibility of the training program, and therefore the sample size was small and not adequately powered to detect differences in competency outcomes between groups. Additionally, participants’ satisfaction and acceptability ratings were generally very positive, suggesting a potential desirability bias. On the other hand, appropriateness was ranked lower, suggesting the need for improvements to the training programs to promote engagement and sustained interest, which was further reflected during the focus group discussions. The self-report measure used to assess competency outcomes was translated into Hindi and adapted to the local context, though the psychometric properties of this measure have not yet been established for use in rural India. It will be important that further efforts seek to validate this self-report competency measure to support its widespread use in diverse contexts in India. While more scalable and efficient to administer, the use of a self-report measure for competency presents other disadvantages compared to conventional competency assessment methods such as role plays or direct observation because it may not capture the application of skills during direct interactions between the health worker and patient. Furthermore, we made conscious efforts to limit potential bias during the quantitative and qualitative data collection. For instance, the quantitative surveys about satisfaction and acceptability with the training programs may have been subject to social desirability bias, where participants may have reported highly positive responses. To minimize this potential risk, members of our research team overseeing data collection were not involved in the intervention development, and they also reassured participants that there are no right or wrong answers to the questions about program satisfaction because honest feedback is most important for finding ways to improve the training program and content for the future. To minimize a similar risk of social desirability bias in the qualitative data collection, we ensured that the facilitators of the focus group discussions and note taking were also not members of our team involved in the training program development, as they may have influenced participants’ responses. Members of our research team who were not involved in the development of the training program conducted the focus group discussions and collected field notes. Given that our study was primarily aimed at informing a subsequent large-scale trial, we did not conduct an in-depth thematic analysis of our qualitative findings. Therefore, in future research developing digital applications, we can build on our approach presented here to strengthen the qualitative methods for analysis and interpretation of participants’ feedback and recommendations about program design.

We made an effort to recruit only participants who had not previously participated in our formative research as a method to minimize prior exposure. However, there is still a possible risk of contamination [71], which we did not assess, though we believe that this risk was low. Furthermore, the non-specialist health workers were recruited from real world settings; therefore, it is not possible to fully minimize contamination in such settings, as health workers may look up information about the training materials on the Internet or may talk to each other about the program content in routine encounters in the workplace. Participants were recruited from three community health centers in a single district in Madhya Pradesh, indicating that these findings may not generalize to other settings in India in terms of context and culture, or other settings globally. However, many of the findings reported here relate to the use of digital training in a low-resource setting and overcoming challenges such as low digital literacy and poor bandwidth likely apply to many other settings. Our finding that some cadres of health workers, such as the MPWs, were not able to complete the training due to their prior engagement with work commitments highlights potential challenges for scaling up this type of training program due to competing priorities. Therefore, our findings may only generalize to health workers who have the time available and who are interested and willing to learn about treatment for depression. To achieve the goal of universal access to mental health services, it will be necessary to consider what cadres of health workers are available and ideally positioned to successfully complete the training program and provide care for depression and other mental disorders as part of their routine service delivery. Furthermore, we restricted our sample to non-specialist health workers with minimum 8th standard education to ensure that they were literate and able to follow the written training materials, to access and navigate the training program on the smartphone app, and to answer the questions on the competency assessment measure. This type of training would likely not be suitable for health workers who may be illiterate, or who may not be able to operate a smartphone. Even though we found that roughly half of the sample had ever used a smartphone, all participants randomized to either of the digital training programs were able to learn to use the smartphone and access the training program content. This further attests to the interest among non-specialist health workers to use digital technology to support their work, which has been consistently reported in prior studies [68,69].

## 5. Conclusions

The findings and observations from this pilot study offer insights that can inform modifications and improvements to the face-to-face and digital training programs for non-specialist health workers in preparation for a larger fully powered effectiveness trial. A potentially important finding from this pilot study was the apparent motivation for enrollment and starting the training on depression care among non-specialist health workers (*n* = 36 out of *n* = 42) and the motivation to complete this training (*n* = 27 out of *n* = 36), and specifically among ASHAs and ASHA Facilitators. Based on our findings, there seems to be a demand for training in depression care that will be further explored in the forthcoming trial. With digital technologies becoming an increasingly important tool in health systems in many low-resource settings in India, as reflected by efforts to finance the adoption of smartphones among frontline health workers to support care delivery [29], future research can expand on the findings reported here to determine how technology can support the scale up of mental health care. Importantly, it will be necessary to determine how to effectively leverage digital technology to enable supervision and quality assurance for the sustained delivery of high quality psychological treatment for depression, as this will be critical to support task-sharing mental health services in low-resource settings towards addressing the care gap [72].

## Figures and Tables

**Table 1:** Baseline socio-demographic characteristics of study participants.

Socio-Demographic Characteristics	F2F *n*	DGT *n*	DGT+ *n*	*p*-Value
	*n* = 14	*n* = 14	*n* = 14	
Gender				1
Female	11 (79%)	11 (79%)	11 (79%)	
Male	3 (21%)	3 (21%)	3 (21%)	
Designation				1
ASHA	8 (57%)	7 (50%)	8 (57%)	
ASHA Facilitator	3 (21%)	4 (29%)	3 (21%)	
MPW	3 (21%)	3 (21%)	3 (21%)	
Education				0.51
8th to 10th	8 (57%)	9 (64%)	6 (43%)	
Above 10th	6 (43%)	5 (36%)	8 (57%)	
Experience in years mean (95% CI) *	9.73 (6.71, 12.76)	8.96 (5.37, 12.55)	8.38 (5.32, 11.44)	0.8153
Age in years mean (95% CI) *	36.07 (31.34, 40.80)	37.71 (32.43, 42.99)	36 (31.31, 40.68)	0.8341
Type of mobile phone owned #				0.931
Ordinary mobile phone	7 (50%)	6 (43%)	6 (43%)	
Smartphone	7 (50%)	7 (50%)	8 (57%)	
Family Size (number of persons in household) mean (95% CI)	5.3 (4.19, 6.42)	4.85 (3.07,6.63)	5.35 (3.62, 7.09)	0.868
Previous Experience in Mental Health Training **	(*n* = 11)	(*n* = 12)	(*n* = 14)	0.591
Yes	5 (46%)	8 (67%)	8 (57%)	
No	6 (54%)	4 (33%)	6 (43%)	
How many years before took part in the training mean (95% CI) *	3 (0.67, 5.32)	3 (2.22, 3.77)	3.13 (1.6, 4.66)	0.9814

# one missing value in DGT arm. * Means ± CIs are presented for continuous variables, counts for categorical variables. ** The non-specialist health workers had previously learned about mental health issues associated with domestic violence during their routine trainings. However, they have not received any formal training on delivering brief psychological treatments for mental health problems such as depression. We collected the data for this question after the baseline assessment; hence, the number of respondents is lower. F2F: Face-to-Face; DGT: Digital Training; ASHA: Accredited Social Health Activists; MPW: Multi-Purpose Workers.

**Table 2:** Summary of process indicators across the three different training programs.

Number of Participants	F2F (*n* = 14)	DGT (*n* = 14)	DGT+ (*n* = 14)
Started the training	10 (71%)	12 (86%)	14 (100%)
Completed the full training (all modules)	8 (57%)	8 (57%)	11 (79%)
Did not complete all of the modules in the training	2 (14%)	4 (29%)	3 (21%)
Number of phone calls made by participants for seeking technical assistance	NA	149	57
Number of phone calls made by the research team to participants for follow up on their queries	NA	106	87

F2F: Face-to-Face; DGT: Digital Training.

**Table 3:** Common technical challenges mentioned by participants during phone calls with the research team in the digital training programs.

Registered Queries by Phone	Specific Technical Challenges Encountered
Internet related	Internet is not working There is no phone connectivity network in the area Internet speed is slow Internet data is over, should I recharge it Course is not opening even connecting on Wi-Fi
Smartphone handling related	How to operate smartphone features Don’t know how to use a smartphone App has been deleted/removed from phone
Moodle Learning Management System app related	Got logged out from course The app is requiring me to enter the password The app is showing an error on the screen Videos are not opening in the app Quizzes are not showing up in the app Videos are running very slow, show continuous booting
Course navigation related	How to attempt quizzes and in which order to attempt them How to erase the wrong answer if entered mistakenly in the assessment quiz How to see in the app how much of the course is completed How to know what grades I have scored Completed all three given attempts but would like to attempt more to increase my scores, how to do it
Smartphone hardware/software related	Phone screen has been broken Phone is lost

**Table 4:** Participant ratings of satisfaction and acceptability with the training programs *.

Domains of Satisfaction and Acceptability (F2F and DGT)	Study Arms		
	F2F (*n* = 11)	DGT (*n* = 12)	DGT+ (*n* = 13)
	Mean (SD)	Mean (SD)	Mean (SD)
**Acceptability**	5.6 (0.44)	5.2 (1.10)	5.5 (0.52)
The instructor was available to answer my questions about the coursework (F2F)./I could find answers to questions I had about the coursework (DGT).	5.6 (0.51)	5.1 (1.16)	5.5 (0.51)
The instructor was willing to assist me if I needed help in the course (F2F)./Answers to questions about the coursework were easy to understand (DGT).	5.4 (0.51)	5.3 (1.40)	5.2 (1.14)
The instructor cared about how well I did in this course (F2F)./The instructor in the recorded lecture cared about helping me to learn (DGT).	5.4 (1.26)	5.3 (1.21)	5.5 (0.66)
The instructor was respectful of me (F2F)./The instructor in the recorded lecture used a respectful tone (DGT).	5.7 (0.48)	5.3 (1.21)	5.5 (0.66)
The instructor was friendly (F2F)./The instructor in the recorded lecture used a friendly tone (DGT).	5.7 (0.48)	4.8 (1.40)	5.2 (1.16)
I believe that the instructor cared about my feelings (F2F)./The recorded lecture used familiar language and expressions (DGT).	5.6 (0.51)	5.4 (1.24)	5.8 (0.6)
**Appropriateness**	4.9 (0.57)	5.0 (0.74)	5.1 (0.64)
The coursework held my attention (F2F/DGT).	5.4 (0.51)	5.3 (0.65)	5.3 (1.10)
The instructional methods used in this course held my attention (F2F/DGT).	4.9 (1.59)	5.0 (1.27)	4.9 (1.38)
I enjoyed the instructional methods used in this course (F2F/DGT).	5.3 (0.48)	5.3 (0.98)	5.5 (0.77)
The instructional methods engaged me in the course (F2F/DGT).	2.7 (1.88)	3.3 (1.82)	4.0 (1.77)
I enjoyed completing the coursework (F2F/DGT).	5.7 (0.48)	5.3 (1.13)	5.5 (0.66)
The coursework was interesting to me (F2F/DGT).	5.5 (0.52)	5.6 (0.66)	5.5 (0.51)
**Adoption**	5.5 (0.38)	5.5 (0.79)	5.6 (0.43)
In general, the coursework was useful to me (F2F/DGT).	5.2 (0.42)	5.5 (0.52)	5.5 (0.51)
The coursework was beneficial to me (F2F/DGT).	5.3 (0.48)	5.7 (0.65)	5.7 (0.48)
I found the coursework to be relevant to my future (F2F/DGT).	5.7 (0.48)	5.4 (0.79)	5.6 (0.50)
I will be able to use the knowledge I gained in this course (F2F/DGT).	5.6 (0.51)	5.4 (1.24)	5.6 (0.50)
The knowledge I gained in this course is important for my future (F2F/DGT).	5.6 (0.51)	5.6 (1.16)	5.6 (0.50)
**Feasibility**	5.2 (0.40)	5.4 (0.81)	5.5 (0.42)
I had the opportunity to decide for myself how to meet the course goals (F2F/DGT).	4.9 (1.10)	5.7 (0.49)	5.8 (0.43)
I was confident that I could succeed in the coursework (F2F/DGT).	5.1 (1.19)	5.3 (1.21)	5.6 (0.50)
I had the freedom to complete the coursework my own way (F2F/DGT).	5.4 (0.51)	5.3 (0.65)	5.8 (0.43)
I felt that I could be successful in meeting the academic challenges in this course (F2F/DGT).	5.3 (0.48)	5.2 (1.19)	5.2 (1.21)
I had options on how to achieve the goals of the course (F2F/DGT).	4.4 (1.26)	5.4 (0.79)	5.2 (1.09)
I was capable of getting a high grade in this course (F2F/DGT).	5.5 (0.52)	5.0 (1.53)	5.5 (0.66)
I had control over how I learned the course content (F2F/DGT).	5.0 (1.15)	5.2 (1.33)	5.5 (0.66)
Throughout the course, I felt that I could be successful on the coursework (F2F/DGT).	5.3 (0.48)	5.4 (1.16)	5.6 (0.51)
I had flexibility in what I was allowed to do in this course (F2F/DGT).	5.5 (0.52)	5.8 (0.38)	5.6 (0.51)

* The satisfaction and acceptability questionnaire was adapted from an existing measure of motivation and engagement in education programs called the MUSIC^®^ model of motivation inventory [55,56,57,58]. The measure consists of 26 items and was tailored to the Face-to-Face (F2F) or digital (DGT) training and translated into Hindi for use in this study. The items are rated on a six-point Likert scale, with 1 being the lowest and 6 the highest score. The questionnaire covers the domains of acceptability, appropriateness, adoption, and feasibility. The average score of each domain was calculated by adding the score of all the items in the domain divided by the number of questions in the domain. F2F: Face-to-Face; DGT: Digital Training.

**Table 5:** Summary of key findings from the focus group discussions with participants in the three training programs.

Focus Group Discussion Themes	F2F (*n* = 8)	DGT (*n* = 9) and DGT+ (*n* = 11)
**Acceptability**		
Facilitators	Became aware about depression; before the training, did not know much about it Felt happy and good about having the chance of being part of training on mental health and depression Pleased with the way trainers conducted the training	Enjoyed the subject of the training and found it useful to help people with stress and tension Able to relate to the training subject as they had experienced symptoms of depression in their own lives or among people around them Pleased to learn about depression and mental illness as it was a new subject for them Learned how to do the training on smartphones Digital training app was easy to understand and navigate Digital training app was attractive, interactive, and well-designed
Challenges and/or suggestions to address the challenges		Some participants sought help from family members (i.e., husband, children, neighbor, etc.) to address challenges faced while using the smartphone and understanding the app
**Appropriateness**		
Facilitators	Trainers taught the course and were able to address participants’ questions The role plays and group activities were helpful to learn the topic and helped to decrease hesitation while performing role plays Participants used training materials to supplement role plays and group activities Liked to learn about the HAP model, PHQ-9, and counseling skills (e.g., how to provide mental support and talk to the person with stress, how to sit during a session, how to ask questions, etc.)	Understandable language Learning through video lectures was helpful for learning the content and more quickly Interesting to learn about symptoms of depression and how to identify them, suicide risks, PHQ-9, and counseling processes and counseling skills (e.g., how to build rapport and how to talk with patients) Provision of a list of all modules was useful Multiple chances for attempting questions was helpful to answer the questions correctly and learn more about particular topics Did not use the help tab given on the digital training app when facing problems, instead phoned to seek support from the research team The support provided by the research team was helpful in addressing questions and challenges Active support from the research team worked as a motivating factor Interactive questions were interesting and kept participants engaged with the course Some participants found the training manual helpful, others did not feel the need to use it
Challenges and/or suggestions to address challenges	Some participants did not use training manual during training The training manual should contain details of all sessions of HAP modules explaining practical guidelines to carry out HAP activities and sessions instead of theoretical HAP details Training was too packed and heavy, felt like a lot of content taught in six days Training days can be increased but that will not be feasible, hence the alternative is to organize refresher training after every 3 or 6 months	Learning PHQ-9 and interpretation of the PHQ-9 score and activity chart was challenging to understand for some participants during the first time viewing the course content; but participants were able to understand the content after reviewing the content again Did not check the notifications and messages sent on the digital training app Found it difficult to comprehend messages on the phone that were in the English language At the time of orientation to the training program, the purpose and use of the help option on the digital training app should be explained Calling hours from 10 am to 5 pm to seek support should be extended as health workers remain busy with their work schedules during these hours Some of the participants suggested to add subtitles to the videos
**Adoption**		
Facilitators	HAP training will be useful to identify the people with stress or tension (i.e., local term for depression) in the community and help them through counseling Training will be useful for health workers to also address their own mental health issues	This training can be helpful in providing counseling to people and especially to pregnant women with stress and tension
Challenges and/or suggestions to address challenges		Wanted the course on their mobile device after completing the training so that they can relearn the training if they forget anything
**Feasibility**		
Facilitators		Convenient and flexible to learn the training in the time allotted Can learn and re-learn the content if needed
Challenges and/or suggestions to address challenges	Some words in the training were difficult to understand	Poor internet connectivity created disturbance in learning, and irritation and sometimes frustration lowered motivation to learn Make the entire course offline to address the issue of poor internet connectivity Poor mobile network in some of the villages Use a different mobile service provider to address connectivity issues Difficulty in understanding how to submit the answers online Include digital orientation training as part of the course to address the technical challenges Deleted the digital training app by mistake Due to the challenge of poor internet connectivity, unable to access all content from the modules as all videos did not play

F2F: Face-to-Face; DGT: Digital Training; HAP: Healthy Activity Program; PHQ-9: 9-item Patient Health Questionnaire.

## References

[1] Sagar R., Dandona R., Gururaj G., Dhaliwal R., Singh A., Ferrari A., Dua T., Ganguli A., Varghese M., Chakma J.K. (2020). The burden of mental disorders across the states of India: The Global Burden of Disease Study 1990–2017. Lancet Psychiatry.

[2] Arvind B.A., Gururaj G., Loganathan S., Amudhan S., Varghese M., Benegal V., Rao G.N., Kokane A.M., Chavan B., Dalal P. (2019). Prevalence and socioeconomic impact of depressive disorders in India: Multisite population-based cross-sectional study. BMJ Open.

[3] Pathare S., Brazinova A., Levav I. (2018). Care gap: A comprehensive measure to quantify unmet needs in mental health. Epidemiol. Psychiatr. Sci..

[4] Patel V., Xiao S., Chen H., Hanna F., Jotheeswaran A., Luo D., Parikh R., Sharma E., Usmani S., Yu Y. (2016). The magnitude of and health system responses to the mental health treatment gap in adults in India and China. Lancet.

[5] Kokane A., Pakhare A., Gururaj G., Varghese M., Benegal V., Rao G.N., Arvind B., Shukla M., Mitra A., Yadav K. (2019). Mental Health Issues in Madhya Pradesh: Insights from National Mental Health Survey of India 2016. Healthcare.

[6] World Health Organization (2016). mhGAP Intervention Guide Mental Health Gap Action Programme Version 2.0 for Mental, Neurological and Substance use Disorders in Non-Specialized Health Settings.

[7] Fairburn C.G., Patel V. (2014). The global dissemination of psychological treatments: A road map for research and practice. Am. J. Psychiatry.

[8] Kazdin A.E. (2017). Addressing the treatment gap: A key challenge for extending evidence-based psychosocial interventions. Behav. Res. Ther..

[9] Raviola G., Naslund J.A., Smith S.L., Patel V. (2019). Innovative Models in Mental Health Delivery Systems: Task Sharing Care with Non-specialist Providers to Close the Mental Health Treatment Gap. Curr. Psychiatry Rep..

[10] Singla D.R., A Kohrt B., Murray L.K., Anand A., Chorpita B.F., Patel V. (2017). Psychological Treatments for the World: Lessons from Low- and Middle-Income Countries. Annu. Rev. Clin. Psychol..

[11] Hoeft T.J., Fortney J.C., Patel V., Unützer J. (2016). Task-Sharing Approaches to Improve Mental Health Care in Rural and Other Low-Resource Settings: A Systematic Review. J. Rural. Heal..

[12] Barbui C., Purgato M., Abdulmalik J., Acarturk C., Eaton J., Gastaldon C., Gureje O., Hanlon C., Jordans M., Lund C. (2020). Efficacy of psychosocial interventions for mental health outcomes in low-income and middle-income countries: An umbrella review. Lancet Psychiatry.

[13] Ministry of Health & Family Welfare (2014). New Pathways New Hope National Mental Health Policy of India.

[14] Ministry of Law and Justice (2017). The Mental Health Care Act 2017.

[15] Directorate General of Health Services (2017). National Mental Health Programme.

[16] Ahuja S., Shidhaye R., Khan A., Roberts T., Jordans M., Thornicroft G., Petersen I. (2020). Understanding mental health system governance in India: Perspectives of key stakeholders. Preprints.

[17] Barnett M.L., Lau A.S., Miranda J. (2018). Lay health worker involvement in evidence-based treatment delivery: A conceptual model to address disparities in care. Annu. Rev. Clin. Psychol..

[18] Barnett M.L., Gonzalez A., Miranda J., Chavira D.A., Lau A.S. (2018). Mobilizing Community Health Workers to Address Mental Health Disparities for Underserved Populations: A Systematic Review. Adm. Policy Ment. Health.

[19] Padmanathan P., De Silva M.J. (2013). The acceptability and feasibility of task-sharing for mental healthcare in low and middle income countries: A systematic review. Soc. Sci. Med..

[20] National Health Mission (2017). Update on ASHA Programme.

[21] Ministry of Health and Family Welfare, Government of India (2011). National Rural Health Mission.

[22] Ved R., Scott K., Gupta G., Ummer O., Singh S., Srivastava A., George A. (2019). How are gender inequalities facing India’s one million ASHAs being addressed? Policy origins and adaptations for the world’s largest all-female community health worker programme. Hum. Resour. Health.

[23] National Rural Health Mission (2011). ASHA: Which way forward? Evaluation of ASHA Programme.

[24] Fairburn C.G., Cooper Z. (2011). Therapist competence, therapy quality, and therapist training. Behav. Res. Ther..

[25] Naslund J.A., Shidhaye R., Patel V. (2019). Digital Technology for Building Capacity of Nonspecialist Health Workers for Task Sharing and Scaling Up Mental Health Care Globally. Harv. Rev. Psychiatry.

[26] Naslund J.A., Aschbrenner K.A., Araya R., Marsch L.A., Unützer J., Patel V., Bartels S.J. (2017). Digital technology for treating and preventing mental disorders in low-income and middle-income countries: A narrative review of the literature. Lancet Psychiatry.

[27] Keelery S. Mobile Phone Internet Users in India 2015–2023. https://www.statista.com/statistics/558610/number-of-mobile-internet-user-in-india/.

[28] Modi D., Dholakia N., Gopalan R., Venkatraman S., Dave K., Shah S., Desai G., Qazi S.A., Sinha A., Pandey R.M. (2019). mHealth intervention “ImTeCHO” to improve delivery of maternal, neonatal, and child care services—A cluster-randomized trial in tribal areas of Gujarat, India. PloS Med..

[29] Saha S., Kotwani P., Pandya A., Patel C., Shah K., Saxena D., Puwar T., Desai S., Patel D.M., Sethuraman A. (2020). Addressing comprehensive primary healthcare in Gujarat through mHealth intervention: Early implementation experience with TeCHO+ programme. J. Fam. Med. Prim. Care.

[30] Rahman A., Akhtar P., Hamdani S.U., Atif N., Nazir H., Uddin I., Nisar A., Huma Z., Maselko J., Sikander S. (2019). Using technology to scale-up training and supervision of community health workers in the psychosocial management of perinatal depression: A non-inferiority, randomized controlled trial. Glob. Ment. Health.

[31] Maulik P.K., Kallakuri S., Devarapalli S., Vadlamani V.K., Jha V., Patel A. (2017). Increasing use of mental health services in remote areas using mobile technology: A pre–post evaluation of the SMART Mental Health project in rural India. J. Glob. Health.

[32] Shields-Zeeman L., Pathare S., Walters B.H., Kapadia-Kundu N., Joag K. (2017). Promoting wellbeing and improving access to mental health care through community champions in rural India: The Atmiyata intervention approach. Int. J. Ment. Health Syst..

[33] Gonsalves P.P., Hodgson E.S., Kumar A., Aurora T., Chandak Y., Sharma R., Michelson D., Patel V. (2019). Design and development of the ‘POD Adventures’ smartphone game: A blended problem-solving intervention for adolescent mental health in India. Front. Public Health.

[34] Muke S., Shrivastava R., Mitchell L., Khan A., Murhar V., Tugnawat D., Shidhaye R., Patel V., Naslund J.A. (2019). Acceptability and feasibility of digital technology for training community health workers to deliver evidence-based psychosocial treatment for depression in rural India. Asian J. Psychiatry.

[35] Patel V., Weobong B., Weiss H.A., Anand A., Bhat B., Katti B., Dimidjian S., Araya R., Hollon S.D., King M. (2017). The Healthy Activity Program (HAP), a lay counsellor-delivered brief psychological treatment for severe depression, in primary care in India: A randomised controlled trial. Lancet.

[36] Homitz D.J., Berge Z.L. (2008). Using e-mentoring to sustain distance training and education. Learn. Organ..

[37] Friedman L.W., Friedman H. (2013). Using social media technologies to enhance online learning. J. Educ. Online.

[38] Eldridge S.M., Chan C.L., Campbell M.J., Bond C.M., Hopewell S., Thabane L., Lancaster G.A. (2016). CONSORT 2010 statement: Extension to randomised pilot and feasibility trials. BMJ.

[39] Directorate of Census Operations Madhya Pradesh (2011). Census of India 2011: Provisional Population Totals Madhya Pradesh.

[40] Suryanarayana M., Agrawal A., Seeta Prabhu K. (2011). Inequality-Adjusted Human Development Index for India’s States.

[41] Menon P., Deolalikar A., Bhaskar A. (2009). India State Hunger Index: Comparisons of Hunger Across States.

[42] Billingham S.A., Whitehead A.L., Julious S.A. (2013). An audit of sample sizes for pilot and feasibility trials being undertaken in the United Kingdom registered in the United Kingdom Clinical Research Network database. BMC Med. Res. Methodol..

[43] National Health Mission (2018). Nation Health Mission (M.P.).

[44] Saprii L., Richards E., Kokho P., Theobald S. (2015). Community health workers in rural India: Analysing the opportunities and challenges Accredited Social Health Activists (ASHAs) face in realising their multiple roles. Hum. Resour. Health.

[45] Scott K., George A.S., Ved R.R. (2019). Taking stock of 10 years of published research on the ASHA programme: Examining India’s national community health worker programme from a health systems perspective. Health Res. Policy Syst..

[46] Satpathy S. (2005). Indian public health standards (IPHS) for community health centres. Indian J. Public Health.

[47] Directorate General of Health Services (2010). Indian Public Health Standard (IPHS) For Community Health Centres (Revised 2010).

[48] Harris P.A., Taylor R., Minor B.L., Elliott V., Fernandez M., O’Neal L., McLeod L., Delacqua G., Delacqua F., Kirby J. (2019). The REDCap consortium: Building an international community of software platform partners. J. Biomed. Inform..

[49] Chowdhary N., Anand A., Dimidjian S., Shinde S., Weobong B., Balaji M., Hollon S.D., Rahman A., Wilson G.T., Verdeli H. (2016). The Healthy Activity Program lay counsellor delivered treatment for severe depression in India: Systematic development and randomised evaluation. Br. J. Psychiatry.

[50] Weobong B., Weiss H.A., McDaid D., Singla D.R., Hollon S.D., Nadkarni A., Park A.-L., Bhat B., Katti B., Anand A. (2017). Sustained effectiveness and cost-effectiveness of the Healthy Activity Programme, a brief psychological treatment for depression delivered by lay counsellors in primary care: 12-month follow-up of a randomised controlled trial. PLoS Med..

[51] Walker I.F., Khanal S., Hicks J.P., Lamichhane B., Thapa A., Elsey H., Baral S.C., Newell J.N. (2018). Implementation of a psychosocial support package for people receiving treatment for multidrug-resistant tuberculosis in Nepal: A feasibility and acceptability study. PLoS ONE.

[52] Jordans M.J., Luitel N.P., Garman E., Kohrt B.A., Rathod S.D., Shrestha P., Komproe I.H., Lund C., Patel V. (2019). Effectiveness of psychological treatments for depression and alcohol use disorder delivered by community-based counsellors: Two pragmatic randomised controlled trials within primary healthcare in Nepal. Br. J. Psychiatry.

[53] Shidhaye R., Baron E., Murhar V., Rathod S., Khan A., Singh A., Shrivastava S., Muke S., Shrivastava R., Lund C. (2019). Community, facility and individual level impact of integrating mental health screening and treatment into the primary healthcare system in Sehore district, Madhya Pradesh, India. BMJ Glob. Health.

[54] Khan A., Shrivastava R., Tugnawat D., Singh A., Dimidjian S., Patel V., Bhan A., Naslund J.A. (2020). Design and Development of a Digital Program for Training Non-specialist Health Workers to Deliver an Evidence-Based Psychological Treatment for Depression in Primary Care in India. J. Technol. Behav. Sci..

[55] Jones B.D. User Guide for Assessing the Components of the MUSIC^®^ Model of Motivation. https://www.themusicmodel.com/.

[56] Jones B.D. (2009). Motivating students to engage in learning: The MUSIC model of academic motivation. International. J. Teach. Learn. High. Educ..

[57] Jones B.D. (2010). An examination of motivation model components in face-to-face and online instruction. Electron. J. Res. Educ. Psychol..

[58] Jones B.D. (2018). Motivating Students by Design: Practical Strategies for Professors.

[59] Cooper Z., Doll H., Bailey-Straebler S., Bohn K., de Vries D., Murphy R., O’Connor M.E., Fairburn C.G. (2017). Assessing therapist competence: Development of a performance-based measure and its comparison with a web-based measure. JMIR Ment. Health.

[60] Restivo J.L., Mitchell L., Joshi U., Anand A., Gugiu P.C., Hollon S.D., Singla D.R., Patel V., Naslund J.A., Cooper Z. (2020). Assessing health worker competence to deliver a brief psychological treatment for depression: Development and validation of a scalable measure. J. Behav. Cogn. Ther..

[61] Popham W.J. (2001). Teaching to the Test?. Educ. Leadersh..

[62] Yardley L., Morrison L., Bradbury K., Muller I. (2015). The person-based approach to intervention development: Application to digital health-related behavior change interventions. J. Med. Internet Res..

[63] Gale N.K., Heath G., Cameron E., Rashid S., Redwood S. (2013). Using the framework method for the analysis of qualitative data in multi-disciplinary health research. BMC Med. Res. Methodol..

[64] Rey D., Neuhäuser M., Gibbons J.D., Chakraborti S. (2011). Wilcoxon-Signed-Rank Test. International Encyclopedia of Statistical Science.

[65] Naslund J.A., Gonsalves P.P., Gruebner O., Pendse S.R., Smith S.L., Sharma A., Raviola G. (2019). Digital Innovations for Global Mental Health: Opportunities for Data Science, Task Sharing, and Early Intervention. Curr. Treat. Options Psychiatry.

[66] Mohr D.C., Lyon A.R., Lattie E.G., Reddy M., Schueller S.M. (2017). Accelerating digital mental health research from early design and creation to successful implementation and sustainment. J. Med. Internet Res..

[67] Merchant R., Torous J., Rodriguez-Villa E., Naslund J.A. (2020). Digital technology for management of severe mental disorders in low-income and middle-income countries. Curr. Opin. Psychiatry.

[68] Winters N., Langer L., Nduku P., Robson J., O’Donovan J., Maulik P., Paton C., Geniets A., Peiris D., Nagraj S. (2019). Using mobile technologies to support the training of community health workers in low-income and middle-income countries: Mapping the evidence. BMJ Glob. Health.

[69] Winters N., Langer L., Geniets A. (2018). Scoping review assessing the evidence used to support the adoption of mobile health (mHealth) technologies for the education and training of community health workers (CHWs) in low-income and middle-income countries. BMJ Open.

[70] Biemba G., Chiluba B., Yeboah-Antwi K., Silavwe V., Lunze K., Mwale R.K., Russpatrick S., Hamer D.H. (2017). A mobile-based community health management information system for community health workers and their supervisors in 2 districts of Zambia. Glob. Health Sci. Pract..

[71] Keogh-Brown M., Bachmann M., Shepstone L., Hewitt C., Howe A., Ramsay C.R., Song F., Miles J., Torgerson D., Miles S. (2007). Contamination in trials of educational interventions. Health Technol. Assess..

[72] Kemp C.G., Petersen I., Bhana A., Rao D. (2019). Supervision of Task-Shared Mental Health Care in Low-Resource Settings: A Commentary on Programmatic Experience. Glob. Health: Sci. Pract..

